# Assessment of Dental Students’ Knowledge and Practice on Root Canal Irrigation

**DOI:** 10.7759/cureus.97840

**Published:** 2025-11-26

**Authors:** Ekin D Catmabacak, Ahmet Kerem Eren

**Affiliations:** 1 Department of Endodontics, Trakya University, Edirne, TUR

**Keywords:** cross sectional studies, dental education, endodontics, root canal disinfection, root canal irrigation protocols, undergraduate curriculum

## Abstract

Introduction: Irrigation is a fundamental phase of root canal treatment, directly influencing disinfection efficacy and treatment prognosis. Understanding how well dental students integrate evidence-based irrigation principles into their clinical practice is essential for improving endodontic education and patient care outcomes.

Method: A cross-sectional survey was administered to 150 undergraduate dental students (79 fourth-year and 71 fifth-year students) at Trakya University. The 25-item questionnaire, validated through expert review, assessed theoretical understanding of irrigants, clinical protocol adherence, activation system usage, complication management, and information-seeking behaviors. Responses were analyzed using descriptive statistics and Chi-square or Fisher’s exact tests, with Bonferroni correction where applicable.

Results: Both groups demonstrated high theoretical knowledge of irrigant properties and interactions, with correct responses recorded for sodium hypochlorite (NaOCl) mechanism, chlorhexidine rationale, ethylenediaminetetraacetic acid (EDTA) purpose, and NaOCl-EDTA interaction. However, only 33 (41.8%) fourth-year and 29 (40.8%) fifth-year students identified the recommended irrigant volume (>20 mL). Fourth-year students more frequently irrigated after every file change (71 (89.9%) vs. 50 (70.4%), p = 0.005), whereas fifth-year students more often reported reducing irrigation duration due to time constraints (27 (38.0%) vs. 15 (19.0%), p = 0.015). Use of activation techniques was low (10 (12.7%) vs. 11 (15.5%)), and reliance on manual irrigation predominated. A significant difference was observed in the use of side-vented or closed-ended needles (65 (82.3%) vs. 43 (60.6%), p = 0.005). Regarding education, most students reported receiving only theoretical training in activation systems (111 (74.0%)), while nearly half of fifth-years (33 (46.5%)) indicated difficulty in designing case-specific protocols compared with fourth-years (18 (22.8%), p = 0.003). Students primarily relied on peer consultation and internet resources for additional information.

Conclusion: While foundational knowledge of irrigants is strong, translation into consistent, evidence-based clinical practice remains limited. Curricular strategies should incorporate structured, hands-on training with activation systems, simulation of complex cases, and integration of guideline-based decision-making frameworks to enhance clinical competence.

## Introduction

Root canal therapy (RCT) success relies not only on the chemical and mechanical effectiveness of root canal disinfection but also on the clinician’s ability to apply evidence-based protocols consistently in daily practice. In undergraduate dental education, the development of these competencies begins with theoretical instruction on irrigants, progresses through preclinical exercises, and culminates in supervised clinical application. However, multiple studies have shown that the transition from theory to practice remains a critical challenge for dental students, especially when managing complex irrigation protocols in real patient scenarios [1-3].

Chemical irrigation is a cornerstone of root canal disinfection, supplementing mechanical instrumentation in removing debris, biofilm, and tissue remnants from anatomically complex areas [4-6]. While sodium hypochlorite (NaOCl) remains the gold standard, adjunctive agents such as chelating agents and antimicrobial solutions are incorporated for specific purposes, each with advantages, limitations, and potential chemical interactions [5-7]. Advanced technologies, such as passive ultrasonic irrigation (PUI), laser-activated irrigation (LAI), and multisonic systems, offer improved smear layer removal and deeper penetration of irrigants [7, 8]. Mastery of these techniques, however, requires both repeated hands-on practice and a clear understanding of their indications and limitations.

In many dental curricula, irrigant properties and protocols are taught in early preclinical years, often without exposure to advanced activation devices until later clinical stages [1, 9]. This sequential approach can result in a gap: students may perform well in knowledge-based assessments but feel less confident when making protocol decisions during complex clinical cases. Limited access to activation systems, variability in supervision, and time constraints further contribute to deviations from ideal practice, even among senior students [2, 3].

Given the increasing complexity of irrigation protocols and the emphasis of recent international guidelines [8] on precise sequencing, activation, and safety measures, it is essential to evaluate how well undergraduate education equips students to translate theoretical knowledge into clinical competence. This cross-sectional survey therefore aims to systematically evaluate fourth- and fifth-year dental students’ knowledge, clinical practices, and decision-making competence regarding root canal irrigation, with particular emphasis on the alignment of their protocols with contemporary evidence-based guidelines and the identification of specific educational gaps that may require curricular improvement.

## Materials and methods

Study design and ethical considerations

This cross-sectional study was conducted at Trakya University. Ethical approval was obtained from the Non-Interventional Scientific Research Ethics Committee, Faculty of Medicine, Trakya University (Approval No.: 10/272, dated May 26, 2025), and all procedures adhered to the principles of the Declaration of Helsinki. Participation was voluntary and anonymous, and no personal identifiers were collected.

Participants and inclusion criteria

The target population consisted of fourth and fifth-year undergraduate dental students. Eligibility criteria included being enrolled in Trakya University Faculty of Dentistry and having completed at least one clinical endodontic internship rotation. Students who did not meet these criteria were excluded from the study. No formal sample size calculation was performed due to the exploratory nature of the study; however, all eligible students were invited to participate to maximize representativeness. During the study period, a total of 190 students were eligible (fourth- and fifth-year combined). A total of 150 students completed the survey (79 fourth-year and 71 fifth-year students).

Questionnaire development and content validation

The questionnaire was developed based on relevant literature and previously validated instruments used in similar studies. Content validity was established through expert review by two academic staff members from the Department of Endodontics. Minor revisions were made to improve clarity and relevance before distribution.

Data collection tool and procedure

A structured, self-administered questionnaire (see Appendices) comprising 25 closed-ended questions was developed to evaluate students’ knowledge, clinical practices, and confidence levels regarding root canal irrigation. The questionnaire included items addressing the theoretical understanding of irrigants and their properties, irrigation protocols and adjunctive techniques, perceived educational adequacy, and information-seeking behavior.

The questionnaire was distributed online via Google Forms (Google LLC, Mountain View, CA, USA) between May 2025 and July 2025, and responses were automatically collected in Microsoft Excel (Microsoft Corp., Redmond, WA, USA) format. It included both single-response questions (such as selecting the best choice or correct mechanism) and multiple-response questions (such as identifying applicable complications, strategies, or methods), and participants were instructed to respond based on their current knowledge and clinical experiences.

The questionnaire was designed such that participants could not proceed to the next item without completing the current one. Therefore, no missing or incomplete responses were recorded, and all submitted data were complete and included in the final analysis.

Informed consent was obtained by virtue of participants completing the survey.

The final questionnaire is organized under thematic subheadings in the Appendix, with original question numbering preserved to maintain consistency with the analyses.

Data analysis

All statistical analyses were performed using IBM SPSS Statistics for Windows, version 30 (IBM Corp., Armonk, NY, USA). A p-value of <0.05 was considered statistically significant. Descriptive statistics were used to summarize frequencies and percentages for categorical variables. Where applicable, 95% confidence intervals were reported.

Comparative analyses were conducted to assess differences between fourth- and fifth-year students in terms of knowledge levels, perceived educational adequacy and access to information, and clinical application practices.

For each multiple-choice question, the observed frequency of responses for each option was compared with the expected frequency, calculated as the total number of responses divided by the number of available options. To identify the most frequently selected options, one-sample chi-square tests were performed for each option to assess whether the observed frequency was significantly higher than the expected value. Given the multiple comparisons within each question, the Bonferroni correction was applied to control the family-wise error rate. Options with Bonferroni-adjusted p-values < 0.05 were considered significantly more frequently selected than expected.

For each option identified as significantly over-selected, additional comparative analyses between fourth- and fifth-year students were conducted using either Pearson’s chi-square test or Fisher’s exact test, depending on the distribution of the observed frequencies. In cases where multiple comparisons were conducted, the Bonferroni correction was applied to control for type I error.

## Results

A total of 150 students participated in the study, comprising 82 female subjects and 68 male subjects. Of these, 79 were fourth-year students, and 71 were fifth-year students (Table 1).

**Table 1:** Demographic characteristics of the participant cohort.

Variable	Category	n
Academic year	Fourth-year	79
	Fifth-year	71
Gender	Female	82
	Male	68
Total		150

Knowledge level assessment: properties and interactions of irrigation solutions

The first group of questions evaluated the students’ theoretical understanding of endodontic irrigation solutions, focusing on their primary properties, mechanisms of action, and possible chemical interactions. Overall, high correct response rates were observed across both academic years, indicating satisfactory theoretical knowledge (Table 2).

**Table 2:** Knowledge of root canal irrigation principles and related procedures. Chi-square tests (Q5, Q7) and Fisher’s exact tests (Q3, Q6) yielded p-values > 0.05, indicating comparable performance across both cohorts. CHX: chlorhexidine; NaOCl: sodium hypochlorite; EDTA: ethylenediaminetetraacetic acid; df: degrees of freedom; χ²: Chi-square.

Question	Fourth year n (%)	Fifth year n (%)	Chi-square (χ²)	df	p-value
Q5: Reason for preferring CHX	70 (88.61%)	61 (85.92%)	0.062	1	0.803
Q7: Outcome of simultaneous use of NaOCl and EDTA	71 (89.87%)	63 (88.73%)	0	1	1
Question	Fourth year n (%)	Fifth year n (%)	Odds ratio	p-value	
Q3: Main mechanism of action of NaOCl	75 (94.94%)	68 (95.77%)	0.83	1	
Q6: Purpose of using EDTA	75 (94.94%)	67 (94.37%)	1.119	1	

For the item assessing the primary mechanism of action of NaOCl (Q3), 75 (94.9%) of fourth-year and 68 (95.8%) of fifth-year students responded correctly, with no statistically significant difference between the groups (Fisher’s exact test, p > 0.05).

Similarly, the question addressing the rationale for using chlorhexidine (CHX) as an auxiliary irrigant (Q5) showed high accuracy, with correct response rates of 70 (88.6%) for fourth-year and 61 (85.9%) for fifth-year students (Chi-square test, p > 0.05).

For the item examining the primary function of EDTA in smear layer removal (Q6), 75 (94.9%) of fourth-year and 67 (94.3%) of fifth-year students answered correctly, again with no significant difference (Fisher’s exact test, p > 0.05).

Finally, the question evaluating knowledge of the chemical interaction between NaOCl and EDTA (Q7) yielded correct response rates of 71 (89.9%) and 63 (88.7%) for fourth- and fifth-year students, respectively, with no statistical difference observed (Chi-square test, p > 0.05).

Clinical practice habits

This section (Q1, Q2, Q4, Q8-Q19, Q21, Q22) examined students’ practical approaches to endodontic irrigation, including irrigant selection and sequencing, application strategies, activation protocols, safety measures, and adaptations for complex cases (Table 3). The analysis also explored potential differences between fourth- and fifth-year students, reflecting the impact of increased clinical exposure on procedural choices.

**Table 3:** Clinical practices and procedural preferences in root canal irrigation. Items marked with an asterisk (*) indicate statistically significant differences between fourth- and fifth-year students (p < 0.05). CHX: chlorhexidine; NaOCl: sodium hypochlorite; EDTA: ethylenediaminetetraacetic acid; df: degrees of freedom; χ²: Chi-square.

Question	Option	Fourth year n (%)	Fifth year n (%)	χ²	df	p-value
Q2: Use of supplementary irrigant solutions	CHX	56 (70.9%)	53 (74.6%)	0.111	1	0.739
	EDTA	62 (78.5%)	58 (81.7%)	0.082	1	0.774
Q4: Measures to enhance NaOCl effectiveness	Activation techniques (ultrasonic/sonic, etc.)	58 (73.4%)	56 (78.9%)	0.348	1	0.555
	Extending the application time	46 (58.2%)	41 (57.7%)	0	1	1.000
Q8: Habit of intermediate rinsing between irrigants	Intermediate rinsing in all irrigant transitions	45 (57.0%)	48 (67.6%)	1.374	1	0.241
Q9: Irrigation practice before canal shaping	Yes, I routinely apply it to reduce microbial load and increase intracanal lubrication.	74 (94.9%)	63 (90%)	0.613	1	0.433
	Yes, I apply it because I believe it can reduce apical debris extrusion.	4 (5.1%)	7 (10%)	0.658	1	0.417
Q10: Consideration of irrigant contact time	Yes, but I only pay attention to the contact time for chelating agents.	30 (38%)	18 (25.4%)	2.188	1	0.556
							I generally determine the irrigation time based on subjective perception; I do not follow a specific time.	16 (20.3%)	24 (33.8%)	2.851	1	0.365
	Q11: Frequency of irrigation during canal shaping	I irrigate after every file change	71 (89.9%)	50 (70.4%)	7.867	1							0.005*
	Q12: Average total irrigant volume per canal	>20 ml	33 (41.8%)	29 (40.8%)	0	1	1.000
Q13: Main clinical factor influencing irrigation protocol	Pulpal and/or periapical condition	72 (91.1%)	59 (83.1%)	1.519	1	0.435
Q14: Description of personal irrigation protocol	I follow the sequence NaOCl → EDTA → saline intermediate rinse → CHX, without using sonic/ultrasonic activation for the irrigants.	23 (29.1%)	18 (25.4%)	0.11	1	0.739
							I follow the sequence NaOCl → EDTA → saline intermediate rinse → CHX, with sonic/ultrasonic activation for the irrigants.	10 (12.7%)	11 (15.5%)	0.069	1	0.791
													After NaOCl irrigation, I perform an intermediate rinse with saline, followed by CHX; irrigation is performed manually.	10 (12.7%)	10 (14.1%)	0	1	0.987
	During shaping, I apply manual irrigation with NaOCl, then use EDTA (17%) as the final irrigant, supported with sonic/ultrasonic activation.	10 (12.7%)	7 (9.9%)	0.079	1	0.777
	During shaping, I apply manual irrigation with NaOCl, then use EDTA (17%) as the final irrigant without activation.	10 (12.7%)	7 (9.9%)	0.079	1	0.777
	Q15: Perceived role of irrigation in treatment success	Irrigation is one of the essential components of endodontic success, and its effect is indispensable.	74 (93.67%)	62 (87.32%)	1.109	1	0.292
	Q16: Reduction of irrigation duration due to time constraints	Rarely such a need arises; I usually maintain my irrigation duration.	40 (50.6%)	26 (36.6%)	2.438	1	0.118
		Yes, I occasionally encounter such situations and reduce the irrigation duration.	15 (19%)	27 (38%)	5.813	1	0.015*
Q17: Preferred irrigation method	I perform only manual irrigation as I do not have access to activation systems.	68 (86.1%)	50 (70.4%)	4.566	1	0.032*
							Q18: Safety measures to prevent apical extrusion	I position the needle tip 1–2 mm shorter than the working length.	77 (97.47%)	70 (98.59%)	0	1	1
I perform irrigation with low pressure and controlled speed.	77 (97.47%)	67 (94.37%)	0.303	1	0.581		
I ensure isolation with a rubber dam.	72 (91.14%)	60 (84.51%)	0.993	1	0.319		
I use side-vented or closed-ended irrigation needles.	65 (82.28%)	43 (60.56%)	7.702	1	0.005*		
I avoid vigorous irrigation before completing canal shaping.	41 (51.9%)	32 (45.07%)	0.451	1	0.501		
Q19: Modifications of irrigation protocol in necrotic/retreatment cases	I apply additional auxiliary irrigants (e.g., EDTA, CHX, citric acid).	61 (77.22%)	60 (84.51%)	0.85	1	0.356
	I extend the irrigation duration (e.g., irrigation after each file, intracanal solution retention, etc.).	55 (69.62%)	51 (71.83%)	0.014	1	0.906
Q21: Main limiting factor in using activation systems	Limited access to relevant devices or systems	53 (67.09%)	48 (67.61%)	0	1	1
Q22: Most frequent problem encountered during irrigation	Lack of access to activation systems (e.g., PUI, sonic, negative pressure)	34 (43.04%)	35 (49.30%)	0.364	1	0.546
	Inadequate irrigation time and volume due to clinical time constraints	21 (26.58%)	18 (25.35%)	0	1	1.000

Irrigant Selection and Sequencing

For the item assessing the primary irrigant solution (Q1), all students in both academic years reported NaOCl as their primary irrigant, confirming its universal acceptance as the gold standard in root canal disinfection.

The question on the use of supplementary irrigant solutions (Q2) showed that EDTA and CHX were the most frequently selected adjuncts, each chosen by more than 70% of students in both groups (EDTA: 62 (78.5%) vs. 58 (81.7%); CHX: 56 (70.9%) vs. 53 (74.6%)), with no statistically significant differences (p > 0.05).

Q8, regarding the habit of intermediate rinsing between irrigants, showed that most students reported rinsing for all transitions (45 (57.0%) of fourth-years; 48 (67.6%) of fifth-years), while smaller proportions limited rinsing to NaOCl-CHX or NaOCl-EDTA transitions. No predominant protocol was identified across participants (p > 0.05).

The question on the description of personal irrigation protocol (Q14) revealed that the two most common protocols were NaOCl → EDTA → saline → CHX, either with or without sonic/ultrasonic activation. Among fourth-years, 23 (29.1%) students preferred the non-activation protocol and 10 (12.7%) used activation; in fifth-years, these rates were 18 (25.4%) and 11 (15.5%), respectively.

Regarding the average total irrigant volume per canal (Q12), 33 (41.8%) of fourth-year and 29 (40.8%) of fifth-year students selected the recommended amount (>20 mL), with no significant difference between the groups (p > 0.05).

Irrigation Timing, Contact Time, and Volume

For the practice of irrigation before canal shaping (Q9), all students reported initiating irrigation prior to instrumentation, albeit for varying reasons, aligning with best-practice recommendations (p > 0.05).

The consideration of irrigant contact time (Q10) showed variation: the most common approach was timing only for chelating agents (30 (38.0%) of fourth-years; 18 (25.4%) of fifth-years), followed by subjective estimation without a fixed protocol.

A statistically significant difference emerged in the frequency of irrigation during shaping (Q11), with 71 (89.9%) of fourth-year versus 50 (70.4%) of fifth-year students irrigating after every file change (p = 0.005).

The impact of clinical constraints on irrigation duration (Q16) was also significant, with 27 (38.0%) of fifth-year students compared with 15 (19.0%) of fourth-year students reporting occasional reduction in irrigation time under pressure (p = 0.015).

Activation Protocols

The question on measures to enhance NaOCl effectiveness (Q4) showed that most students reported using activation techniques (58 (73.4%) vs. 56 (78.9%)) or extending application time (46 (58.2%) vs. 41 (57.7%)), with no significant differences (p > 0.05).

For the preferred irrigation method (Q17), a significantly higher proportion of fourth-year students reported performing only manual irrigation due to lack of access to activation systems (68 (86.1%) vs. 50 (70.4%), p = 0.032).

The main limiting factor for activation system use (Q21) was limited access to devices (53 (67.1%) vs. 48 (67.6%), p > 0.05). The most frequent problems during irrigation (Q22) were lack of access to activation systems (34 (43.0%) vs. 35 (49.3%)) and inadequate time/volume due to clinical constraints (21 (26.6%) vs. 18 (25.4%)), without significant group differences (p > 0.05).

Safety Measures

Q18, assessing safety measures to prevent apical extrusion, revealed a significant difference in the use of side-vented or closed-ended needles (65 (82.3%) vs. 43 (60.6%), p = 0.006), while other preventive measures (e.g., needle positioning, pressure control, rubber dam use) showed no significant differences between groups.

For the main clinical factor influencing irrigation protocol (Q13), most students prioritized pulpal or periapical condition (72 (91.1%) vs. 59 (83.1%), p > 0.05).

The perceived role of irrigation in treatment success (Q15) was confirmed by the majority, with 74 (93.7%) of fourth-year and 62 (87.3%) of fifth-year students strongly agreeing on its indispensability (p > 0.05).

In the modification of irrigation protocol for necrotic or retreatment cases (Q19), the most common changes were extending irrigation time (55 (69.6%) vs. 51 (71.8%)) and adding irrigants (61 (77.2%) vs. 60 (84.5%)), with no significant differences between the two cohorts (p > 0.05).

Evaluation of educational content and information-seeking behaviors

This section examined the adequacy of undergraduate endodontic education in preparing students for clinical irrigation practice (Table 4). The training received on irrigation activation systems (Q20) showed that the majority of students reported receiving only theoretical instruction, with no significant difference between fourth- and fifth-year cohorts (60 (75.9%) vs. 51 (71.8%), p > 0.05).

**Table 4:** Educational experience, perceived deficiencies, and coping strategies. Items marked with an asterisk (*) indicate statistically significant differences between fourth- and fifth-year students (p < 0.05). CHX: chlorhexidine; NaOCl: sodium hypochlorite; EDTA: ethylenediaminetetraacetic acid; df: degrees of freedom; χ²: Chi-square.

Question	Option	Fourth year n (%)	Fifth year n (%)	χ²	df	p-value
Q20: Training received on irrigation activation systems during undergraduate education	Yes, only theoretical information was provided	60 (75.95%)	51 (71.83%)	0.150	1	0.698
Q23: Areas of self-perceived deficiency in irrigation knowledge or practice	Recent developments in new generation irrigants and irrigation technologies	68 (86.08%)	58 (81.69%)	0.259	1	0.611
	Indications, technical aspects, and effectiveness of irrigation activation methods	38 (48.10%)	35 (49.30%)	0	1	1
	Ability to design clinical irrigation protocols and make case-based decisions	18 (22.78%)	33 (46.48%)	8.329	1	0.003*
Q24: Coverage of irrigation protocols and complication management during training	Irrigation was presented comprehensively and systematically; theoretical instruction was supported with hands-on training	36 (45.57%)	35 (49.30%)	0.086	1	0.7698
	Basic irrigation concepts (e.g., NaOCl, EDTA use) were explained but activation systems and complications were insufficiently addressed	29 (36.71%)	25 (35.21%)	0.000	1	0.983
Q25: Preferred sources of information on irrigation	Consulting colleagues / clinical senior advisors	60 (75.95%)	61 (85.92%)	1.785	1	0.181
	My previous notes/student materials	57 (72.15%)	41 (57.75%)	2.820	1	0.093
	Internet search engines (e.g., Google, YouTube)	49 (62.03%)	42 (59.15%)	0.037	1	0.847

The areas of self-perceived deficiency (Q23) revealed broadly similar patterns between groups. No significant differences were observed for knowledge of recent developments in irrigants (68 (86.1%) vs. 58 (81.7%), p > 0.05) or the technical aspects of activation methods (38 (48.1%) vs. 35 (49.3%), p > 0.05). However, a significantly greater proportion of fifth-year students reported difficulty in designing clinical irrigation protocols and making case-based decisions (33 (46.5%) vs. 18 (22.8%), p = 0.003).

For the coverage of irrigation protocols and complication management during training (Q24), both cohorts reported similar rates of comprehensive systematic instruction supported by hands-on practice (36 (45.6%) vs. 35 (49.3%), p > 0.05), and in receiving only basic information without adequate coverage of activation systems or complications (29 (36.7%) vs. 25 (35.2%), p > 0.05).

Regarding the preferred sources of information (Q25), both groups relied most heavily on peer consultation (60 (75.9%) vs. 61 (85.9%), p > 0.05) and internet search engines (49 (62.0%) vs. 42 (59.2%), p > 0.05). Fourth-year students more frequently reported using their own notes and student materials (57 (72.2%) vs. 41 (57.8%), p > 0.05), though this difference was not statistically significant.

Overall, findings suggest consistency in educational exposure between the two academic years, with the main exception being greater difficulty among fifth-year students in clinical decision-making for irrigation protocols, reflecting their increased exposure to complex clinical scenarios.

## Discussion

Root canal irrigation demands not only theoretical understanding but also confident clinical execution, and this study demonstrates that many students remain underprepared for this complexity despite formal instruction.

At Trakya University, irrigation concepts are introduced in the second year at a theoretical level; however, because the preclinical curriculum at this stage is largely devoted to cavity preparation, students have no opportunity for active practice of irrigation procedures. In the third year, hands-on training in root canal irrigation is introduced during preclinical endodontics, when students begin shaping procedures. These sessions are limited to manual irrigation, as activation systems are not available in the preclinical setting.

Clinical training begins in the fourth year, when students start treating patients under supervision. Initially, they are assigned relatively simple cases, most often single-rooted, single-canal teeth, with limited molar treatments. By the fifth year, students are expected to manage more complex endodontic cases, including a higher proportion of molar treatments and retreatment procedures, thereby facing greater anatomical and procedural variability. In the clinical setting, both sonic and ultrasonic activation systems are available, and their use is actively encouraged.

The results indicated that both fourth- and fifth-year students possessed a generally high level of theoretical knowledge, particularly regarding the basic properties and chemical interactions of commonly used irrigants such as NaOCl, CHX, and EDTA. Correct response rates for these items consistently exceeded 85% (e.g., NaOCl mechanism of action (Q3: 75 (94.9%) vs. 68 (95.8%)); CHX rationale (Q5: 70 (88.6%) vs. 61 (85.9%)); EDTA purpose (Q6: 75 (94.9%) vs. 67 (94.3%)); NaOCl-EDTA interaction (Q7: 71 (89.9%) vs. 63 (88.7%))) with no statistically significant differences between academic years. These findings suggest that the foundational principles of endodontic irrigation are being effectively delivered through the current curriculum. However, high scores in knowledge-based questions do not necessarily translate into clinical proficiency, as reflected in students’ reported uncertainties in case-based decision-making and protocol design.

Furthermore, although EDTA was selected more frequently than CHX, it was marked by only 62 (78.5%) fourth-year and 58 (81.7%) fifth-year participants, despite being an essential component of the correct irrigation protocol. While its higher selection rate compared to CHX reflects awareness of smear layer removal and the role of chelating agents in optimizing canal cleanliness before obturation, the fact that not all students selected EDTA indicates a persistent gap in protocol adherence [10]. For the use of supplementary irrigants (Q2), a considerable proportion of students reported using CHX as an adjunct irrigant (56 (70.9%) vs. 53 (74.6%)), demonstrating awareness of its antimicrobial potential. However, despite its historical use and continued preference among some students, CHX is notably absent from the 2025 European Society of Endodontology (ESE) S3-level clinical recommendations, consistent with American Associations of Endodontists (AAE) guidance [8, 11]. This omission likely reflects a shift in clinical consensus, de-emphasizing CHX due to its lack of tissue-dissolving ability and risk of harmful precipitate formation with NaOCl [10, 12]. The continued student reliance on CHX may therefore highlight a knowledge gap regarding current evidence-based irrigation strategies [1, 13].

Effective irrigation relies not only on the choice of irrigant but also on its timing, frequency, and volume during chemomechanical preparation. Responses to items on irrigation practice before canal shaping (Q9), consideration of irrigant contact time (Q10), frequency of irrigation during canal shaping (Q11), and average total irrigant volume per canal (Q12) provide an integrated view of students’ knowledge and application of irrigation protocols. For irrigation timing (Q9), 74 (94.9%) of fourth-year and 63 (90.0%) of fifth-year students reported initiating irrigation before canal instrumentation, consistent with recommendations that stress early chemical disinfection to reduce microbial load and aid debris removal [14]. This suggests that foundational knowledge regarding the initiation of irrigation is generally well internalized across both academic levels.

However, when examining the monitoring of irrigant contact time (Q10), a more fragmented pattern emerged. While 30 (38.0%) of fourth-year students and 18 (25.4%) of fifth-year students reported paying attention to the contact time of chelating agents such as EDTA, only 16 (20.3%) and 24 (33.8%), respectively, indicated monitoring contact time for all irrigants. A significant proportion relied on subjective estimation without following specific timing protocols, reflecting only a partial understanding of the chemical debridement dynamics critical to endodontic success. In clinical practice, effective biofilm disruption requires adequate contact between irrigants and contaminated dentin [10, 11]. Although NaOCl acts rapidly, organic matter and tissue remnants can reduce its efficacy, making continuous irrigation with fresh solution and adequate contact time essential.

A statistically significant difference emerged in frequency of irrigation during shaping (Q11), with 71 (89.9%) of fourth-year students irrigating after every file change compared to 50 (70.4%) of fifth-year students (p = 0.005). This inverse trend may reflect procedural drift, reduced supervision, or time constraints in advanced years. Given that frequent irrigation is essential to prevent apical packing and maintain debris suspension [15], the decline raises concerns about sustaining foundational protocols. As students gain autonomy, they may prioritize efficiency over procedural accuracy, particularly in time-constrained clinical settings, a trend supported by the finding in reduction of irrigation duration due to time constraints (Q16), where 27 (38.0%) of fifth-year students more frequently admitted to reducing irrigation duration compared with 15 (19.0%) of fourth-year students (p = 0.015), as discussed later in this section. Q12 assessed awareness of the recommended irrigant volume per root canal system.

The awareness of the recommended irrigant volume per root canal system (Q12) showed that 33 (41.8%) of fourth-year and 29 (40.8%) of fifth-year students selected the correct range (>20 mL). However, many reported using less or not measuring it consciously, indicating underestimation of its role in disinfection. Insufficient volume can impair debris removal, shorten chemical contact time, and reduce efficacy, particularly in the apical third. These responses point to a potential underestimation of irrigant volume as a critical determinant of disinfection efficacy.

The description of personal irrigation protocols (Q14) reported by students showed substantial variability, with no single approach adopted by the majority. The most common sequence (NaOCl → EDTA → saline rinse → CHX without activation) was selected by 23 (29.1%) of fourth-year and 18 (25.4%) of fifth-year students; however, this deviates from the ESE S3-level clinical guidelines, which recommend NaOCl (1-5.25%) followed by 17% EDTA, and optionally a final NaOCl rinse, as the core irrigants for non-surgical root canal treatment [8]. As previously noted, CHX is no longer recommended in current ESE and AAE guidelines. Thus, this choice is inconsistent with evidence-based protocols [8, 11].

From a chemical interaction standpoint, both guidelines emphasize that NaOCl should be used throughout cleaning and shaping for organic tissue dissolution, whereas EDTA is indicated for smear layer removal in the final stages. They further caution that NaOCl and EDTA should not be used simultaneously, as EDTA reduces NaOCl’s tissue-dissolving effect, and NaOCl used after EDTA can cause dentin erosion [8, 11]. The habit of intermediate rinsing between irrigants (Q8)-frequently reported by students, with 45 (57.0%) of fourth-year students and 48 (67.6%) of fifth-year students rinsing in all irrigant transitions-suggests some procedural awareness of chemical compatibility. However, the high variability and partial misalignment with evidence-based protocols point to gaps in understanding the rationale behind irrigant sequencing. This reinforces the need for undergraduate training to integrate not only the what but also the why of irrigation protocols, ensuring that students’ clinical choices are both procedurally correct and scientifically justified.

Only a small proportion of students reported using irrigant activation, despite its well-documented advantages in enhancing debridement and penetration in anatomically complex root canal systems [8]. Reported usage ranged from 10 (12.7%) to 11 (15.5%), indicating limited integration of this evidence-based adjunct into clinical routines. Previous studies have noted that many preclinical protocols fail to replicate clinical reality, particularly in irrigant flow dynamics and activation efficacy, and that inconsistent implementation of advanced irrigation training remains a global issue [1, 13]. This disparity, with activation systems absent in preclinical training but available in clinical practice, may contribute to the reduced confidence and procedural uncertainty observed among students, particularly in applying activation techniques effectively.

Despite similar access to activation devices in clinical settings, many students continued to rely on manual irrigation (Q17: preferred irrigation method), with a significant difference between years (68 (86.1%) of fourth-year students vs. 50 (70.4%) of fifth-year students, p = 0.032), possibly reflecting reduced confidence or perceived time constraints. Notably, while 10 (12.7%) of fourth-year students and 11 (15.5%) of fifth-year students claimed in Q14 (description of personal irrigation protocol) to use activation techniques, 78% also reported in Q17 lacking access to such systems and relying solely on manual irrigation. This apparent contradiction may stem from a tendency among students to select the most “correct” or ideal-sounding response in earlier questions, possibly due to perceived evaluative pressure.

Regarding safety measures to prevent apical extrusion (Q18), although most students used strategies such as short needle positioning and low pressure, only 43 (60.6%) of fifth-year students used side-vented or closed-ended needles compared with 65 (82.3%) of fourth-year students (p = 0.005), indicating reduced adherence to strict protocols with increasing case complexity or workload.

Although over 90% of students acknowledged the role of irrigation in treatment success (Q15) (74 (93.7%) vs. 62 (87.3%), p > 0.05), their reported behaviors revealed a gap between knowledge and practice. In Q16 (reduction of irrigation duration due to time constraints), 27 (38.0%) of fifth-year students admitted to shortening irrigation duration due to time constraints, compared with 15 (19.0%) of fourth-year students (p = 0.015). This suggests that, despite strong theoretical endorsement, real-world pressures such as patient cooperation and limited appointment time may compromise protocol adherence. As clinical responsibilities increase, students may deviate from ideal practices even while recognizing their importance [3].

In Q19 (modifications of irrigation protocol in necrotic/retreatment cases), both cohorts most frequently reported extending irrigation duration (55 (69.6%) vs. 51 (71.8%)) and incorporating additional irrigants (61 (77.2%) vs. 60 (84.5%)). While these adaptations are in line with recommendations to enhance debridement in heavily contaminated canals, the lack of significant differences between academic years suggests that such modifications may be applied more as routine habits than as case-specific, evidence-based decisions.

The combined responses to Q20-Q22 (training, limiting factors, and frequent problems related to activation systems) indicate systemic and educational barriers to the clinical use of irrigation activation systems. Most students reported receiving only theoretical instruction on activation techniques (Q20: 60 (75.9%) vs. 51 (71.8%), p > 0.05), with little or no hands-on experience, contributing to low procedural confidence. In Q21 (main limiting factor in using activation systems), the primary reason was lack of access to devices (53 (67.1%) vs. 48 (67.6%), p > 0.05), followed by insufficient knowledge. Similarly, Q22 (most frequent problem encountered during irrigation) highlighted limited access (34 (43.0%) vs. 35 (49.3%)) and time constraints (21 (26.6%) vs. 18 (25.4%)), with no significant differences between groups. Given that activation systems are available in the clinical setting and their use is actively encouraged, this response is unexpected, suggesting possible underutilization of existing resources or misperceptions regarding their accessibility. Without comprehensive hands-on training and effective integration of these technologies into routine practice, students may continue to rely on suboptimal techniques despite being aware of best practices.

In Q23 (self-perceived deficiencies in designing clinical irrigation protocols and case-based decisions), a significantly larger proportion of fifth-year students (33 (46.5%)) reported difficulty compared with fourth-year students (18 (22.8%), p = 0.003), despite both groups reporting similar levels of formal instruction in irrigation principles. This likely reflects the transition from the controlled preclinical environment to the complexity of real patient care, where anatomical variability, case difficulty, and retreatment scenarios demand more nuanced decision-making. This finding aligns with the “confidence paradox” described in the literature, whereby senior students-despite receiving more advanced training-may feel less prepared as increased exposure to unpredictable clinical situations heightens awareness of their own limitations [1, 3].

A similar pattern emerged in responses related to training on irrigation activation systems (Q20) and coverage of irrigation protocols in undergraduate education (Q24). While most students reported receiving only theoretical instruction (Q20: 60 (75.9%) vs. 51 (71.8%), p > 0.05), fewer felt the training was comprehensive or supported by adequate practical experience (Q24: 36 (45.6%) vs. 35 (49.3%), p > 0.05). Many noted that activation protocols and complication management were underemphasized or difficult to integrate into clinical workflows, mirroring the broader challenge of translating knowledge into confident clinical execution. Such findings highlight the need for curricula that reinforce foundational skills through repeated hands-on practice and structured clinical simulations, ensuring that theoretical competence is matched by procedural proficiency. Similar trends have been reported in the literature, where endodontic irrigation training across dental schools is often variable and fragmented, with limited standardization in the inclusion of activation or complication management modules [9].

In addressing educational gaps, students primarily relied on preferred sources of information (Q25) such as peer consultation (60 (75.9%) vs. 61 (85.9%), p > 0.05) and internet-based resources (49 (62.0%) vs. 42 (59.2%), p > 0.05), indicating a decentralized, self-directed learning approach. While this resourcefulness is positive, heavy dependence on unvetted online content raises concerns about misinformation, particularly regarding irrigant interactions and protocol sequencing. The relatively low use of academic or institution-provided materials may point to accessibility challenges or limited confidence in the clarity and applicability of existing resources. These findings support the need for better-integrated, clinically oriented educational tools, such as decision-making frameworks, flowcharts, and interactive simulations that link theoretical knowledge to procedural execution.

Overall, the findings of this study indicate that, while undergraduate dental students demonstrate solid foundational knowledge of irrigants and their properties, certain areas, such as the use of activation, irrigant sequencing, volume control, and protocol adherence, could benefit from further reinforcement. Addressing factors such as the persistence of outdated habits, limited integration of available clinical technologies, and reliance on subjective decision-making may help bridge the gap between theoretical instruction and clinical application. Enhancing structured, hands-on experiences that simulate clinical complexity, alongside case-based learning and active faculty feedback, could further strengthen students’ readiness to meet the procedural demands of modern endodontic care in line with current international guidelines.

The methodological strengths of this study include its clear and systematic design, the expert-validated survey instrument, and the robust statistical comparison between fourth- and fifth-year students. These elements increased internal consistency and enabled a meaningful evaluation of educational differences in irrigation practices. However, this study has several limitations. First, because it was conducted in a single dental school, the generalizability of the findings to other institutions is inherently limited. Second, no formal a priori sample size calculation was performed, which may reduce the statistical power of the analyses and introduce the risk of non-response bias. Third, the reliance on self-reported questionnaire data may be subject to recall bias or social desirability bias, potentially affecting the accuracy of the responses. Fourth, the study did not include objective clinical performance measures, and therefore, the findings reflect perceived or self-reported behavior rather than direct observation of clinical competence. Finally, although the total number of eligible students (n = 190) has now been reported, the absence of a full population-level denominator for broader external comparisons limits the precision of response rate interpretation. Despite these limitations, the study provides a detailed evaluation of undergraduate irrigation practices in the context of ESE S3-level and AAE guidelines, identifying key deficiencies such as limited activation use, inconsistent contact time monitoring, and under-recognition of recommended irrigant volumes. These findings offer actionable insights for curriculum planning and targeted interventions, supporting the development of graduates equipped to meet the procedural demands of modern endodontic care.

## Conclusions

This cross-sectional study revealed that while undergraduate dental students demonstrate strong theoretical knowledge of endodontic irrigants, certain gaps remain in the clinical application of evidence-based protocols, particularly in areas such as irrigant sequencing, activation system usage, contact time monitoring, and recommended volume adherence. Occasional reliance on traditional approaches and limited use of available activation technologies suggest opportunities for further enhancement of clinical skills training. Strengthening hands-on practice, integrating simulation-based learning, and reinforcing guideline-based protocols may help bridge the gap between theory and practice. Aligning undergraduate education more closely with contemporary international guidelines and offering repeated opportunities for case-based application can further support graduates in managing the procedural complexities of modern endodontic care.

## Appendices

Questionnaire

(The questionnaire consists of a total of 27 items. Section 1 includes two items collecting demographic information, while Sections 2-4 comprise 25 closed-ended items designed to assess students’ knowledge and clinical practice related to root canal irrigation.)

Section 1: Demographic Information

Please indicate your year of study: o Fourth year o Fifth year

What is your gender? o Female o Male o Prefer not to disclose

Section 2: Knowledge Level Assessment

Question 3 - Which of the following is the primary mechanism of action of sodium hypochlorite (NaOCl) as a root canal irrigant solution?

o Creating a chemical barrier on the canal dentin surface to prevent microbial penetration o Binding to dentinal tubules to provide long-term antimicrobial substantivity o Chelating calcium ions to facilitate the removal of inorganic structures o Disinfecting through the dissolution of organic tissues and oxidative degradation of cellular proteins o Removing the smear layer from canal walls to expose the dentinal tubules

Question 5 - Which of the following is one of the main reasons for preferring chlorhexidine gluconate (CHX) as an irrigant solution in endodontic treatment protocols?

o Ability to dissolve organic tissue remnants inside the canal o Capability to remove the smear layer o Providing long-term antimicrobial effect (substantivity) on the dentin surface o Exhibiting a synergistic effect when used with sodium hypochlorite o Chelating calcium ions in root canal dentin

Question 6 - What is the primary purpose of using ethylenediaminetetraacetic acid (EDTA) as an irrigant solution in the root canal system?

o Directly reducing the bacterial load within the canal o Removing the inorganic smear layer from the dentin surface to expose the tubules o Dissolving necrotic pulp and organic debris to achieve disinfection o Providing long-term antimicrobial substantivity on the canal walls o Physically enlarging the dentin walls

Question 7 - Which of the following is the most likely outcome when sodium hypochlorite (NaOCl) and ethylenediaminetetraacetic acid (EDTA) irrigant solutions are applied simultaneously?

o Both solutions’ antimicrobial effects increase, producing a synergistic effect o EDTA reduces the free chlorine content of NaOCl, decreasing its oxidative efficacy o NaOCl enhances the chelating capacity of EDTA o The combination increases dentin surface hardness o No clinically significant interaction is observed between these two agents

Section 3: Clinical Practice Habits

Question 1 - Which of the following agents do you most frequently use as the primary irrigant solution in root canal treatments?

o Sodium hypochlorite (NaOCl) o Chlorhexidine gluconate (CHX) o Ethylenediaminetetraacetic acid (EDTA) o Saline

Question 2 - In your clinical irrigation protocol, which of the following do you use as an adjunctive irrigant solution in addition to the primary irrigant?

o Chlorhexidine gluconate (CHX) o Ethylenediaminetetraacetic acid (EDTA) o MTAD (a mixture of citric acid, detergent, and doxycycline) o QMix (a combination of CHX and EDTA) o Saline or distilled water (for intermediate rinsing or transition between irrigants) o None; I complete the treatment using only the primary irrigant

Question 4 - Which of the following methods do you routinely use to enhance the clinical efficacy of sodium hypochlorite (NaOCl) as a root canal irrigant solution? (You may select more than one option.)

o Increasing the concentration o Heating the solution before application o Supporting with activation methods (ultrasonic/sonic) inside the canal o Allowing the irrigant to remain inside the canal for a specified time o Prolonging the application time o Injecting the solution into the canal at a slow rate

Question 8 - In clinical situations where you apply different irrigant solutions sequentially, what is your habit regarding intermediate rinsing (e.g., with saline or distilled water) to prevent chemical interactions?

o I routinely perform intermediate rinsing during all irrigant transitions o I perform intermediate rinsing only when combining NaOCl and CHX o I perform intermediate rinsing during both NaOCl-CHX and NaOCl-EDTA transitions o I generally do not perform intermediate rinsing and use the irrigants directly in sequence o I am not aware of why intermediate rinsing is necessary

Question 9 - What is your practice regarding the use of an irrigant solution before starting canal shaping? (Please select the option that best reflects your clinical protocol.)

o Yes, I routinely do so to reduce the microbial load and enhance intracanal lubrication o Yes, I do so because I believe it can reduce apical debris extrusion o No, I only initiate irrigation after completing the shaping procedure o I do not consider this practice necessary

Question 10 - During root canal irrigation, do you take into account the contact time (duration of retention) of irrigant solutions inside the canal? (Please select the option that best reflects your clinical practice.)

o Yes, I apply it with a preplanned contact time to enhance the chemical efficacy of the irrigant o Yes, but I only pay attention to the contact time for agents such as EDTA o I usually determine the irrigation time subjectively, without specific time tracking o I do not measure contact time and continue irrigation simultaneously with the shaping process

Question 11 - During the root canal shaping process, which of the following best describes the frequency with which you apply irrigation? (Please select the option that best reflects your personal clinical protocol.)

o I irrigate after each file change o I irrigate once every 2-3 file changes during shaping o I irrigate only when I observe conditions such as debris accumulation, bleeding, or irrigant cloudiness o I do not perform active irrigation during shaping; I irrigate only at the end of the procedure o I do not have a specific protocol for the frequency of irrigation during shaping

Question 12 - During irrigation of a root canal system, what is the average total volume of irrigant you use? (Please respond to include all irrigants used, such as NaOCl, EDTA, etc.)

o Less than 5 ml o Between 5-10 ml o Between 10-20 ml o More than 20 ml o I do not consciously measure the volume used

Question 13 - In your root canal irrigation protocol (e.g., choice of irrigant, concentration, application time, or activation method), which of the following is the most influential clinical factor? (Please select the factor that most affects your irrigation approach in routine clinical practice.)

o Pulpal and/or periapical status (vitality, necrosis, presence of apical lesion, etc.) o Canal morphology and anatomical challenges (e.g., narrow, curved, calcified, C-shaped canals) o Presence of complications (e.g., instrument fracture, apical constriction, suspected perforation) o Clinical symptoms (acute presentations such as severe pain, abscess, swelling) o External factors such as time management, patient cooperation, or duration of the procedure o Personal practice habits and adherence to a routine protocol

Question 14 - Which of the following best describes the irrigation protocol you adopt during clinical practice? (Please consider your irrigation sequence, types of irrigants used, activation techniques, and your preference for customization according to the clinical situation.)

o I perform manual irrigation with NaOCl throughout the shaping process, then use EDTA (17%) as a final irrigant, supported by sonic/ultrasonic activation (e.g., PUI, EndoActivator) o After NaOCl irrigation, I perform intermediate rinsing with saline followed by CHX; irrigation is performed manually o I perform manual irrigation with NaOCl throughout the shaping process, then use EDTA (17%) as a final irrigant without activation o I follow the sequence NaOCl → EDTA → intermediate rinsing with saline → CHX and activate the irrigants with sonic/ultrasonic activation o I follow the sequence NaOCl → EDTA → intermediate rinsing with saline → CHX without activating the irrigants o I apply the NaOCl → saline → CHX protocol (without using EDTA) with manual irrigation o I apply the NaOCl → EDTA → CHX sequence without intermediate rinsing and without using an activation system o I apply the EDTA → CHX sequence without intermediate rinsing and perform irrigation only manually o I perform sequential irrigation with NaOCl → saline → CHX, followed by EDTA, without using an activation system o I use only NaOCl and EDTA, without CHX; irrigants are used manually or with activation depending on the case o I perform intermediate irrigation with EDTA during shaping, followed by final irrigation with NaOCl, using manual irrigation

Question 15 - How do you evaluate the role of root canal irrigation in the long-term success of endodontic treatment? (Please consider the effects of irrigation on microbial debridement, complication prevention, and tissue dissolution.)

o Irrigation is one of the essential components of endodontic success, and its effect is indispensable o Its efficacy is high but is not applied adequately according to standards in clinical practice o Its effect is moderate compared to shaping and obturation o Its effect is limited; success is more related to mechanical shaping and root canal filling o I have no clear opinion / I am unsure

Question 16 - In your clinical practice, do you ever have to shorten your irrigation time due to treatment time constraints (e.g., insufficient time, patient noncompliance)? (Please consider the total irrigation time, contact time, or activation time when responding.)

o Yes, I frequently shorten the irrigation time due to time constraints o Yes, I occasionally encounter such situations and reduce the irrigation time o Rarely, such a need arises; I usually maintain my irrigation time o No, I apply the irrigation time according to my standard clinical protocol in all cases

Question 17 - Which method do you generally prefer when performing root canal irrigation in your clinical practice?

o I use activation systems (e.g., ultrasonic, sonic, XP-Endo Finisher, etc.) as a complement to manual irrigation o I perform all irrigation solely with activation systems (e.g., closed systems such as GentleWave, EndoVac) o I variably combine manual irrigation and activation systems depending on the clinical characteristics of the case o I perform only manual irrigation because I do not have access to activation systems

Question 18 - During endodontic treatment, which of the following measures do you take in your clinical practice to prevent apical extrusion of irrigants and enhance patient safety? (You may select more than one option.)

o I provide isolation with a rubber dam o I use side-vented or closed-end irrigation needles o I position the needle tip 1-2 mm short of the working length o I perform irrigation at low pressure and controlled flow rate o I check the irrigant volume and pressure before applying activation o I avoid forceful irrigation before completing canal shaping o If apical pathology is present, I reduce the irrigation time and volume o In open-apex cases, I prefer a low-concentration irrigant (e.g., 0.5-1% NaOCl) o I keep the activation time short or avoid activation altogether o I perform irrigation using a small volume o I do not take any specific clinical precautions in this regard

Question 19 - In cases with necrotic pulp or requiring retreatment, which of the following modifications do you make to your irrigation protocol? (You may select more than one option.)

o I increase the sodium hypochlorite (NaOCl) concentration (e.g., from 2.5% to 5.25%) o I extend the irrigation time (e.g., after each file, allowing the solution to remain in the canal) o I use sonic or ultrasonic activation systems (e.g., PUI, EndoActivator) o I additionally apply adjunctive irrigants (e.g., EDTA, CHX, citric acid) o I extend the final irrigation time (e.g., ≥1 minute contact with EDTA) o I prefer heated NaOCl (thermal activation) o I direct irrigation using negative-pressure systems (e.g., EndoVac) o I do not make any changes to my irrigation protocol in such cases

Question 21 - Which primary factor most limits your frequency of using endodontic irrigation activation systems?

o High cost of activation systems o Limited access to the relevant devices or systems o Lack of sufficient theoretical or practical knowledge about activation methods o Lack of evidence-based confidence in the clinical efficacy of these systems o Activation prolongs treatment time o Time pressure due to patient load and limited appointment durations o Inadequate teaching of these systems during undergraduate or postgraduate education

Question 22 - Which of the following is the most frequent problem you encounter related to endodontic irrigation in your clinical practice? (Please select only the most frequent problem.)

o Extrusion of irrigants beyond the apical foramen (e.g., apical extrusion, hypochlorite accident) o Postoperative pain and tenderness after endodontic treatment o Lack of access to activation systems (e.g., PUI, sonic, negative pressure) o Risk of chemical interaction between irrigants (e.g., NaOCl-CHX) o Inadequate hands-on training on irrigation during undergraduate education o Difficulty accessing updated irrigation protocols or literature o Inadequate duration and volume of irrigation due to clinical time constraints o Failure to integrate the irrigation step into the endodontic treatment protocol or its omission

Section 4: Evaluation of Educational Content and Information-Seeking Behaviors

Question 20 - During your undergraduate education, did you receive training on endodontic irrigation activation systems (e.g., passive ultrasonic, sonic, negative pressure, etc.)?

o Yes, I received both theoretical (lecture or seminar) and clinical/practical training o Yes, only theoretical information was provided (e.g., lecture, presentation, article review) o Yes, the systems were demonstrated, but I did not perform individual application o No, I did not receive any training on these systems o I do not remember / I am unsure

Question 23 - In which area(s) do you feel most deficient in terms of knowledge, application, or decision-making? (You may select more than one option.)

o Knowledge of the chemical interactions between different irrigant solutions o Indications, technical details, and efficacy of irrigation activation methods o Clinical management of irrigation-related complications (e.g., hypochlorite accident) o Current developments regarding new-generation irrigants and irrigation technologies o Ability to prepare a clinical irrigation protocol and make case-based decisions o I do not feel deficient in any of these areas

Question 24 - During your clinical endodontic education, to what extent were topics such as irrigation protocols, types of irrigants, activation methods, and complication management covered? (Please consider the scope of the educational content and its applicability to clinical practice.)

o Irrigation was comprehensively and systematically covered; theoretical knowledge was supported by practical applications o Basic irrigation information (e.g., use of NaOCl, EDTA) was provided, but activation systems and complications were not sufficiently addressed o Only superficial information was given; irrigation protocol and clinical integration were not adequately covered o Irrigation was addressed so minimally during training that it is barely memorable / not emphasized o I received education on this topic but am unsure how to translate it into clinical practice

Question 25 - When you feel the need for further education on endodontic irrigation or similar clinical topics, which sources do you primarily consult to obtain information? (You may select more than one option.)

o Current scientific publications (PubMed, academic journals, systematic reviews, etc.) o Internet search engines (e.g., Google, YouTube) o Colleagues / clinical senior consultation o Online professional courses and seminar platforms o University / official academic institutions or association resources (e.g., TDB, ESE, AAE) o My previous notes / student materials o Social media channels (Instagram, Facebook, Reddit, Twitter) o Artificial intelligence-based platforms (e.g., ChatGPT, Claude, Bing AI, Perplexity)
